# Evaluating the Utility of a Statewide Pharmacist-Led Psychotropic Medicines Information Service: A Mixed-Methods Study

**DOI:** 10.1007/s10597-026-01619-4

**Published:** 2026-04-20

**Authors:** Viandro Borja, Liza Hopkins, Tosin Olaluwoye, Michael Olasoji

**Affiliations:** 1 Mental and Addiction Health, Alfred Health, Melbourne, Australia; 2 Institute of Health and Wellbeing, Federation University, Ballarat, Australia

**Keywords:** Psychotropic, Medicines information, Drug information, Statewide service, Mental health

## Abstract

Psychotropic medicines are essential in managing mental illness but are associated with complex challenges, including off-label use, polypharmacy, drug interactions, adverse effects, and diverse safety profiles. Effective decision-making requires access to reliable, evidence-based, and context-specific medicines information. Medicines Information (MI) services, particularly those led by pharmacists, provide tailored, evidence-informed advice that supports safe prescribing and optimises patient outcomes.

This study aimed to evaluate the Psychotropic Drug Advisory Service (PDAS) at Alfred Health, a statewide pharmacist-led clinical advisory service. The evaluation sought to (a) determine the impact of advice on patient care, clinical outcomes, and medication safety; (b) assess the usefulness of advice and its uptake by enquirers; and (c) explore user experiences and satisfaction to inform future service development.

A mixed-methods service evaluation was conducted. Quantitative data were collected through a retrospective audit of all enquiries recorded over a 12-month period (January–December 2024). Qualitative data were obtained through semi-structured interviews conducted between March and August 2025 with a total of 17 clinicians, consumers, and carers who had accessed PDAS during the evaluation period. Interviews explored perceptions of service utility, impact on clinical decision-making, and areas for improvement, and were thematically analysed.

PDAS responded to 266 enquiries over the 12-month evaluation period, with enquiries originating from a broad range of clinicians, consumers, and carers across primary, secondary, and community care. Enquiries frequently involved complex psychotropic decision-making; most commonly relating to administration and dosage, choice of medication, adverse effects, drug–drug interactions, and antipsychotic or antidepressant use. Most enquiries were addressed promptly, with the majority receiving an initial response within one hour. Qualitative analysis identified four key themes: *enhancing confidence in clinical decision-making*,* improving access to specialist psychotropic advice*,* facilitating collaborative problem solving*,* and delivering credible*,* evidence-based guidance;* highlighting PDAS’s role as a trusted, accessible, and specialist support service within complex mental health settings. These findings support the utility of a statewide, pharmacist-led psychotropic medicines information service as an important component of contemporary mental health systems, particularly in settings characterised by clinical complexity and/or limited access to specialist expertise.

## Introduction

Psychotropic medicines are integral to the management of mental illness but represent a diverse and high-risk group of medications frequently associated with off-label use, polypharmacy, drug–drug interactions, and broad side effect profiles. As mental health presentations become increasingly complex, so too does the pharmacological management required. This is compounded by the fact that mental health consumers often experience poorer physical health outcomes and higher rates of comorbid medical conditions, which can influence medication choice, safety, monitoring requirements, and response to treatment (Fiorillo & Sartorius, 2021; Chan et al., 2023; Lambert et al., 2017). Challenges such as medication adherence, the need for individualised care, and treatment decisions in vulnerable populations – including children, older adults, and pregnant women – further complicate prescribing. Managing medications for these consumers safely and effectively necessitates access to contemporary, evidence-based medicines information, clinical expertise, and a deep understanding of patient-specific factors. In this context, timely, specialised, and informed decision support is essential to optimise outcomes and minimise risk.

Medicines Information (MI) services, particularly those delivered by pharmacists, are well established as a valuable source of support for clinicians navigating complex medication decisions. Evaluations of pharmacist-led MI services have demonstrated positive impacts on clinical decision-making, patient safety, and therapeutic outcomes (Hands et al., 2002; McEntee et al., 2010; Rutter & Rutter, 2019). A systematic review by Hands et al. (2002) demonstrated that drug information services were associated with improvements in patient care and treatment outcomes, including modification or discontinuation of inappropriate therapies.

Existing evaluations of pharmacist-led medicines information services have largely focused on generalist or hospital-based models across a range of clinical settings (Hands et al., 2002; McEntee et al., 2010; Rutter & Rutter, 2019). More recently, Bramley et al. (2019) demonstrated the value of pharmacist-led medicines helplines in improving patient understanding, satisfaction, and medicines safety outcomes. However, there remains limited evaluation of medicines information services that are specifically focused on psychotropic medicines. Given the complexity, risk profile, and contextual challenges associated with psychotropic prescribing, targeted evaluation of specialist psychotropic MI services is warranted.

Multiple studies have also highlighted the clinical utility and cost-effectiveness of medicines information services. For example, McEntee et al. (2010) reported that over 90% of healthcare professionals incorporated MI advice into clinical decision-making, with many deferring patients care until expert guidance was received. Similarly, Rutter and Rutter (2019) found that MI services supported evidence-informed decisions by assisting clinicians to manage risk and uncertainty in complex clinical situations.

Despite advances in digital medicines information platforms, there is limited evidence that access to these tools alone can reliably support complex, patient-specific psychotropic decision-making, underscoring the importance of expert interpretation and contextualised advice (Bramley et al., 2009; Prior, 2019). While medicines information is widely available from numerous sources, the quality and reliability of this information can vary (Bergmo et al., 2023; Chan et al., 2020; Raban et al., 2016; Battineni et al., 2020). Assessing relevance, accuracy, and credibility is therefore essential to ensure the information is of high quality. Yet access to objective, high-quality psychotropic medicines information remains inconsistent. Many resources require paid subscriptions, and not all sources are current or reliable (Prior, 2019). For example, individual medication product information may lack detail on off-label use, paediatric dosing or obstetric risks, leading to potential misinterpretation. Moreover, newer agents may have incomplete safety data, while information for older medications may be outdated (Albano et al., 2021). The challenge lies not only in locating accurate and unbiased information, but also in translating this information into patient-specific, practical recommendations. Complex clinical dilemmas cannot always be resolved through information retrieval alone; expert interpretation is often required (Albano et al., 2021; Prior, 2019).

In Australia, the value of hospital-based pharmacist-led MI services is well recognised. Evidence suggests enquirers and service users of these medicines’ information services report a high level of satisfaction and positive impact on patient care (Bramley et al., 2019). However, as noted by Prior (2019), several state-based services have been restructured or closed entirely, leaving substantial gaps in clinical support for mental health and primary care professionals. This reduction in access is occurring against a backdrop of increasing clinical complexity, greater need for individualised prescribing advice, and disparities in resource access across healthcare sectors (Campbell et al., 2021). These challenges are further amplified in rural and regional areas, where clinicians often have limited access to in-house specialist support, increasing reliance on external MI services for timely, evidence-informed guidance.

In the mental health setting, access to reliable psychotropic medicines information plays an important role but remains under-evaluated. Tran and Castle (2012) demonstrated the benefits of structured medication information programs in improving patient understanding and adherence in psychiatric settings. Bramley et al. (2019) showed that medicines helplines in hospital settings significantly improved patient outcomes and safety, and Albano et al. (2021) confirmed the clinical value of pharmaceutical medical information services in supporting prescribing decisions and patient education. More recently, Campbell et al. (2021) highlighted the ongoing challenges general practitioners face in sourcing timely, trusted medicines information, especially in complex mental health cases.

Findings from a recent international survey highlighted the information needs and preferences of both healthcare professionals and consumers when making treatment decisions. Health professionals reported that medical information supported their prescribing decisions, informed patient discussions, and assisted with education activities. Meanwhile, consumers noted that access to reliable information enhanced their understanding, boosted confidence in treatment discussions, and improved willingness to commence therapy—further reinforcing the role of accessible, high-quality medicines information services in supporting therapeutic outcomes (Fung et al., 2019).

The Psychotropic Drug Advisory Service (PDAS) at Alfred Health in Victoria, Australia, is a pharmacist-led, statewide medicines information and clinical advisory service that provides evidence-informed advice on psychotropic medication use to a broad customer base across Victoria, including psychiatrists, general practitioners, generalist pharmacists, other health professionals, consumers, carers, and their supporters. Since its inception, PDAS has aimed to support safe and high-quality prescribing practices in mental health. The aim of the present study is to evaluate the service’s utility, value, and future directions using a mixed-methods approach, contributing novel insights into the role of specialised MI services in mental health systems, particularly in the context of rising psychotropic prescribing trends.

The purpose of this evaluation is to determine the utility of the current service as well as identify areas for improvement. The evaluation has three specific aims:

- to describe service utilisation and the characteristics of enquiries received by PDAS over the evaluation period.
- to explore the perceived impact of PDAS advice on patient care and medication safety, including how advice was used by enquiries.
- to understand user experiences, acceptability, and satisfaction with the overall service to inform future service development.

## Methods

### Study Design

This was a mixed-methods, service evaluation project incorporating both quantitative and qualitative data collection methods. The project was reviewed and approved as a low-risk study by the Alfred Health Ethics Committee (Project Number: 121/25).

### Setting

The evaluation was conducted at Alfred Health, a major metropolitan health service in Victoria, Australia. PDAS provides tailored, evidence-informed psychotropic medication advice to a broad range of stakeholders, including healthcare professionals across public and private mental health services, general hospitals, primary care, and community settings, as well as to consumers and carers seeking support with complex medication-related concerns.

PDAS is delivered by pharmacists with advanced training and substantial clinical experience in mental health and psychotropic medicines, typically at senior or specialist practice levels, and embedded within the broader mental health pharmacy service. Pharmacists contributing to PDAS also hold concurrent clinical roles across inpatient, community, and specialist mental health settings, ensuring advice is grounded in contemporary clinical practice. While the service was initially established as a single-clinician model, it now operates within a team-based framework supported by peer review and clinical governance processes, particularly for complex enquiries requiring higher levels of clinical judgement. Enquiries are documented using a standardised medicines information management system, supporting consistent advice, timely responses, and the application of clinical expertise across the majority of enquiries.

### Quantitative Data Collection

PDAS is based at Alfred Health, and the service maintains a publicly accessible website outlining scope and contact details and accepts enquiries via telephone and/or email during business hours. Awareness is supported through regular multidisciplinary education sessions, presentations, and workshops delivered across hospital, community, and primary care settings. PDAS maintains established partnerships with key stakeholders, consumer and carer organisations, Primary Health Networks, and various professional groups. In addition, PDAS engages in ongoing outreach, communication with external partners and participation in professional forums. The service has implemented targeted strategies to build statewide visibility, including network outreach, education delivery, and strengthening links with internal and external stakeholders.

All PDAS enquiries are recorded in a secure medicines information enquiry management system (MiDatabank). A retrospective audit of de-identified enquiries made to PDAS over a 12-month period (Jan 2024 – Dec 2024) was performed. Data extracted and analysed included:

- Enquiry origin (e.g. primary care, secondary care).
- Service user demographics (e.g. medical practitioner, pharmacist, consumer/carer).
- Method of contact/reply (phone, in-person, email).
- Complexity of enquiry (Level 1–3 classification).
- Number/type of resources used to respond to enquiry.
- Time taken to respond to the enquiry.
- Core clinical category or categories addressed (e.g. choice of therapy, indications/contraindications, pharmacokinetics, drug interactions, adverse effects, use in breastfeeding or pregnancy).
- Specific psychotropic medication(s) involved, if applicable.
- Psychotropic drug class(es) related to the enquiry.

Some of the variables were combined and recoded for ease of data analysis and interpretation. The service user demographic, which initially comprised 11 response categories, was reduced to six. Nurses and allied health professionals were merged into a single category, while community pharmacists and hospital pharmacists were recoded to a new “pharmacist” category. Carers and consumers were also recoded as the “consumer” category, while ED consultants, hospital psychiatrists, and hospital registrars were merged into a “hospital medical practitioner” category. Response time is the interval between the enquirer’s contact and the commencement of the response. It was grouped into five categories for the convenience of analysis. The time taken to complete the enquiry, defined as the interval between the commencement of the response and the complete resolution of the enquiry, was categorised in 30-minute intervals up to 120 minutes, with a separate category for durations exceeding 120 minutes. Response time was categorised into 5 groups: “within an hour”, “one to four hours”, “four hours to a day”, “one to two days”, and “above 2 days”.

Descriptive statistical methods were used to summarise the frequency, distribution, and characteristics of enquiries and to identify key patterns in service utilisation. Because the chi-square test criteria were not met, Fisher’s exact test was used to assess the association between complexity and the time taken to complete enquiries, and the relationship between service user demographics and medication class.

## Qualitative Data Collection

Semi-structured interviews were conducted using an interview guide (Appendix 1) with a purposive sample of all PDAS users, including pharmacists, psychiatrists, and consumers/carers, to explore experiences and perceptions of service utility, decision-making impact, and areas for improvement. All enquirers who contacted the PDAS during the data collection period March – August 2025 were invited to participate in a short telephone or video conferenced semi-structured interview at the time of their contact with PDAS.

Participants who indicated that they were interested in taking part (*n* = 25, representing 10% of enquirers), were contacted by a member of the research team and provided with the opportunity to learn more about the project, provide consent and arrange the interview at a time and modality that suited them. Interviews were able to be conducted with 17 people who had contacted PDAS. Interviews were conducted by telephone, audio recorded for accuracy, transcribed verbatim using secure transcription software in Microsoft 365, manually checked for accuracy and thematically analysed (Braun & Clarke, 2019).

### Participants

Interview participants included three consumers, three carers (one father and two mothers), five psychiatrists (three in public practice and two in private practice), four hospital pharmacists (one internal and three from external health services), and two others (one counsellor in a public drug and alcohol service and one member of a peer support group).

## Findings

### Quantitative Findings

The service responded to 266 enquiries over the 12-month period (Jan 2024 – Dec 2024). Most enquiries were received via telephone or email. As shown in Table 1, the majority of enquiries (55.6%) originated from secondary care services (hospital-based) - including mental health registrars, psychiatrists, and pharmacists, with the remainder from primary care or community settings. Consumers and carers represented 19.5% of enquiries. Enquiries most often related to administration and dosage (42.5%), choice or selection of therapy and indications or contraindications (42.1%). A substantial proportion also focused on mental health–related issues (42.1%) as enquirers often sought psychotropic medicines advice that required interpretation within a mental health context, where understanding of diagnosis, symptom profile, or managing mental health deterioration was necessary to inform medication choice, selection, or optimisation. In some enquiries, particularly from consumers and carers, this also involved navigating relevant mental health services or referral pathways alongside provision of medicines advice. Enquirers also frequently sought information on drug–drug interactions (21.1%), and adverse effects (31.6%). As individual enquiries frequently addressed more than one issue, more than one category was frequently assigned; accordingly, percentages are not mutually exclusive and exceed 100%. The most commonly discussed medication classes were antipsychotics (42.9%) and antidepressants (37.7%).

Just over half of enquiries (51.1%) were classified as level 1 complexity, representing straightforward, fact-based queries, while a further 47.0% were classified as level 2 complexity. Enquiries were categorised using the UK Medicines Information Enquiry Level Guidance framework, in which higher levels reflect increasing clinical complexity, integration of multiple information sources, and greater reliance on professional judgement (UK Medicines Information North West Medicines Information Centre, 2010) Table 2.

**Table 1:** Characteristics of enquiries

Characteristics	*n*	(%)
Internal/external enquiry		
External	206	(74.4)
Internal (e.g. Alfred Health and partners)	60	(22.6)
Origin of enquiry		
Secondary care (e.g. Hospital Pharmacist, Registrar, Psychiatrist)	148	(55.6)
Private (member of the public – consumers, carers)	52	(19.6)
Other (support organisations, not specified etc.)	41	(15.4)
Primary care (e.g. GP or Community Pharmacist)	25	(9.4)
Service user demographics		
Medical practitioner (including, psychiatrists, registrars etc.)	88	(33.1)
Pharmacist/pharmacy staff	56	(21.1)
Consumer/Carer	52	(19.5)
Nurses/Allied Health staff	38	(14.3)
General Practitioners	16	(6.0)
Other (includes other health professionals, researchers, not specified etc.)	16	(6.0)
Complexity of enquiry		
Level 1	136	(51.1)
Level 2	125	(47.0)
Level 3	5	(1.9)
Category / Type of enquiry		
Administration and dosage	113	(42.5)
Choice of therapy/indication/contraindication	112	(42.1)
Mental Health and Substance misuse	112	(42.1)
Non-clinical and other	90	(33.8)
Adverse effects and Toxicity	86	(32.3)
Drug-drug interactions	56	(21.1)
Pharmaceutical and pharmacokinetic issues	55	(20.7)
Special populations – e.g. medicines use in renal disease, breastfeeding, pregnancy	17	(6.4)
Availability & supply	8	(3.0)

**Table 2:** Characteristics of the response

Resources utilised to formulate response		
Own clinical knowledge	214	(80.5)
Guidelines and Databases	143	(53.8)
MIMS Online	122	(45.9)
Other sources (e.g. E-sources, internet search, journal articles)	95	(35.7)
Australian Medicines Handbook (AMH)	85	(32.0)
Past enquiries	37	(13.9)
eTG (Therapeutic Guidelines)	32	(12.0)
Alfred Choice & Medication portal^**a**^	16	(6.0)
Colleagues	10	(3.8)
Psychotropic Drug Directory	13	(4.9)
Class of medication		
Antipsychotics	114	(42.9)
Antidepressants	63	(23.7)
Drugs for sleep – sedative/hypnotic	26	(9.8)
Mood Stabilizer	25	(9.4)
Others: Non-psychiatric medications	22	(8.3)
General enquiries about multiple psychotropics	17	(6.4)
Psychostimulants	16	(6.0)
Response time		
Within an hour	164	(61.7)
1 to 4 h	77	(29.0)
4 h to a day	16	(6.1)
1–2 days	8	(3.0)
Greater than 2 days	1	(0.4)
Time taken to complete the enquiry		
Up to 30 minutes	66	(24.8)
31 to 60 minutes	158	(59.4)
61 to 90 minutes	26	(9.8)
91 to 120 minutes	8	(3.0)
Greater than 2 h	8	(3.0)

^**a**^Choice and Medication© is a subscription-based psychotropic medicines information portal that provides consumer-focused psychotropic medicine leaflets and resources for patients, carers, families and healthcare staff.

The most commonly used resource in responding to enquiries was the pharmacist’s own clinical knowledge (80.5%), followed by guidelines and databases (53.8%) and MIMS (45.9%); whereas colleagues were consulted in 3.8% of cases. As multiple resources were typically consulted for a single enquiry, the percentages are not mutually exclusive and therefore exceed 100%.

Most enquiries (61.7%) received an initial response within one hour, with fewer than 5% taking longer than a day to be addressed. In terms of total completion time, approximately one-quarter (24.8%) were finalised within 30 minutes, while nearly three-fifths (59.4%) were completed within 30–60 minutes.

As shown in Table 3, enquiries classified as *level 1 complexity* were significantly more likely to be completed within 30 minutes (44.9%, *p* < 0.001), whereas those of *level 3 complexity* were most often completed after more than two hours (20.0%, *p* < 0.001). This should be interpreted with caution, given the low frequency in the “above 2 hours” category.

**Table 3:** Complexity vs. time taken

	Time Taken to Complete										
	Up to 30 min		31 to 60 min		61 to 90 min		91 to 120 min		Above 2 h		p-value
	n	(%)	n	(%)	n	(%)	n	(%)	n	(%)	< 0.001
Level 1	61	(44.9)	73	(53.7)	1	(0.7)	0	(0.0)	1	(0.7)	
Level 2	5	(4.0)	84	(67.2)	22	(17.6)	8	(6.4)	6	(4.8)	
Level 3	0	(0.0)	1	(20.0)	3	(60.0)	0	(0.0)	1	(20.0)	

As shown in Table 4, pharmacists (57.1%, *p* = 0.001) were statistically significantly the most likely service users to enquire about antipsychotics, followed by hospital medical practitioners (50.0%, *p* = 0.001). General practitioners were significantly more likely to enquire about antidepressants (68.8%, p = 0.014), while nurses and allied health professionals most often sought information about sedative / hypnotics (26.3%, p < 0.001).

**Table 4:** Relationship between demographics and the medication class

Medication class	Demographics												
	Consumer/carer		Hospital medical practitioner		General practitioner (primary care)		Nurses /allied health staff		Pharmacist /pharmacy staff		Other		
	n	(%)	n	(%)	n	(%)	n	(%)	n	(%)	n	(%)	p-value
Antipsychotics	22	(42.3)	44	(50.0)	5	(31.2)	9	(23.7)	32	(57.1)	2	(12.5)	0.001
Antidepressants	10	(19.2)	22	(25.0)	11	(68.8)	11	(29.0)	9	(16.1)	0	(0.0)	0.014
Sedative/Hypnotic	9	(17.3)	1	(1.1)	0	(0.0)	10	(26.3)	5	(8.9)	1	(6.3)	<0.001
Mood Stabilisers	4	(7.7)	15	(17.1)	0	(0.0)	1	(2.6)	4	(7.1)	1	(6.3)	0.259
Other (non-psychiatric)	6	(13.5)	4	(4.6)	2	(12.5)	5	(13.2)	4	(7.1)	0	(0.0)	0.275
Psychostimulants	7	(11.5)	6	(6.8)	1	(6.3)	2	(5.3)	1	(1.8)	0	(0.0)	0.273
General Psychotropics enquiries	4	(7.7)	8	(9.1)	0	(0.0)	4	(10.5)	0	(0.0)	1	(6.3)	0.118

### Qualitative Findings

A total of 17 PDAS users consented to participate in semi-structured interviews, including clinicians from multiple professional backgrounds as well as consumers and carers. All interviews were audio-recorded with consent and transcribed verbatim prior to analysis. Thematic analysis revealed four interrelated themes capturing the service’s impact and perceived value: building confidence in clinical decision-making, addressing gaps in access to specialist advice, facilitating collaborative problem-solving, and delivering credible, evidence-based guidance. Together, these themes illustrate PDAS’s multifaceted role as both an expert medicines information resource and a form of decision support that assists clinicians, consumers and carers to navigate complex psychotropic treatment decisions.

### Building Confidence in Clinical Decision-Making

Across user groups, PDAS was described as a trusted, expert source that instilled confidence in navigating complex or uncertain psychotropic decisions. Participants often referred to the service as a valued “second opinion” that validated their reasoning and reduced anxiety associated with treatment choices. Consumers and carers described increased confidence and a sense of control over their treatment decisions:

“Phoning your service was just part of getting some reassurance that we were doing the right thing… the person on your side was very helpful to sound that out to. Because we’re not doctors.” (P11, Carer).

“It’s helped with my autonomy… and also helped with relieving anxieties associated with my treatment.” (P16, Consumer).

“I was a little bit awkward at first because I wasn’t sure what the exact purpose of the phone number was… but the person I spoke to was very helpful, very knowledgeable, and was able to allay the concerns that I had.” (P12, Carer).

“It sort of gives you a bit of guidance with what to choose and where to go… it’s not a decision, but it’s very helpful.” (P2, Clinician).

Clinicians particularly valued PDAS for affirming decisions around polypharmacy, off-label prescribing, or rare adverse effects, while consumers and carers emphasised the sense of safety and trust it provided. The credibility of PDAS as a pharmacist-led specialist service distinguished it from generic online resources, reinforcing confidence that treatment choices were evidence-informed and appropriate.

### Addressing Gaps in Access to Specialist Advice

Participants frequently identified PDAS as an essential bridge where specialist mental health medicines information was otherwise unavailable—particularly in rural or primary care settings lacking senior or specialist input. The service provided an accessible and responsive expert resource for those practising in isolation or navigating fragmented systems:

“At the moment I’m a mental health pharmacist at [rural health service] and there wasn’t one before me… I don’t have a senior here to refer to, so I’ve used PDAS a lot and they’ve really helped in that way.” (P6, Clinician).

“The GP is great but he’s only got five minutes… I needed that bit of extra support.” (P14, Consumer).

“In private, I guess I’m a contractor, so very much on my own. So it’s really in my own knowledge and what’s on my fingers.” (P4, Clinician).

“The GP wasn’t sure and couldn’t get hold of the psychiatrist for some time, so I contacted the service and was delighted to receive a response within hours.” (P8, Carer).

PDAS was viewed as a professional “safety net,” offering timely, tailored advice when other supports were limited. This theme underscored the value of statewide, cross-sector access to psychotropic expertise, particularly for clinicians managing complex patients outside tertiary centres.

### Facilitating Collaborative Problem-Solving

Participants consistently highlighted the relational and dialogic nature of their interactions with PDAS. Beyond information provision, the service created opportunities for reflective dialogue, enabling users to “think aloud,” clarify uncertainties, and explore alternative treatment pathways collaboratively. While collaboration with clinicians often involved shared clinical reasoning around diagnosis, medication selection and medication optimisation, collaboration with consumers and carers involved deliberation about treatment options, weighing risks and benefits, and jointly navigating next steps:

“It’s good to be able to kind of have a bit of a discussion to articulate things in person.” (P13, Clinician).

“Most of the time they were able to, if not give me a good answer to carry on with, then a good direction to take my question further… they were able to give me an angle to think about the question a bit better.” (P5, Clinician).

“The person I spoke to was very helpful, very knowledgeable…I decided to go ahead and start her [my daughter] on the medication based on the conversation that we had.” (P12, Carer).

“I just had a complex patient that had a lot of polypharmacy and there was a particular question in regard to medication changes.” (P17, Clinician).

“So, I basically used PDAS just for advice when I was trying to kind of adjust my medications…around titrations and side effects, but mainly the titration side of things.” (P14, Consumer).

Participants described how enquiries that initially appeared straightforward often evolved into more complex discussions once the broader clinical context was explored. Through structured questioning or clarification, PDAS pharmacists identified additional considerations that were not immediately apparent. Clinicians noted that what began as a simple dosing or medication adjustment query occasionally required deeper interpretation to support safe and appropriate decision-making. This sense of collaboration distinguished PDAS from other resources, positioning it as an interactive, human-centred model of clinical decision support. The discussions fostered shared understanding and joint discussions between pharmacists and enquirers, enhancing confidence in managing complex or ambiguous scenarios around psychotropic medicines.

### Delivering Credible, Evidence-Based, User-Friendly Guidance

Interviewees valued the depth, clarity, and reliability of information provided by PDAS. Responses were described as comprehensive, well-referenced, and accompanied by relevant evidence, guidelines, or patient-friendly materials that could be shared with colleagues or consumers:

“The fact that they attach articles and evidence as well — I can forward that on to the psychiatrist and they really love that.” (P6, Clinician).

“It’s always very clear and professional, but able to be understood by people with no pharmacological background.” (P8, Carer).

“Yes, yes, it was quite precise with additional [detail]… it opened another path so that if you want to read more, this information may be useful” (P2, Clinician).

“It’s been really helpful and really quick responses as well as really in-depth, which has been really helpful to orient ourselves to the information, especially when we’re knowledgeable on one medication but have to consider all this other medication and how they interact.” (P10, Clinician).

“It wasn’t overly scientific — easy for me to understand because I’m not from a medical background.” (P14, Consumer).

By integrating scholarly resources and clinical expertise, PDAS enabled enquirers to justify treatment decisions, promote shared decision-making, and strengthen continuity of care. The service’s emphasis on practical, evidence-informed communication enhanced both professional credibility and patient understanding.

## Discussion

The overall aim of this study was to evaluate the Psychotropic Drug Advisory Service (PDAS). This mixed-methods evaluation demonstrates that the PDAS provides a distinctive and valuable form of medicines information support across Victoria, Australia. To the best of our knowledge there have been no studies to date that has evaluated a pharmacy led psychotropic drug advisory service. Quantitative findings showed high utilisation from diverse user groups—psychiatrists, pharmacists, general practitioners, and consumers—with most enquiries involving complex psychotropic decisions related to administration and dosage, choice of therapy and indications or contraindications, adverse effects, and drug–drug interactions.

The volume of enquiries observed in this evaluation (266 enquiries over 12 months) is consistent with, and comparable to, activity reported for PDAS in recent years. Service data indicate that PDAS typically manages between approximately 200 and 275 enquiries annually, with the most recent reporting period reflecting a 37% increase in demand compared with the preceding year. While most published evaluations of pharmacist-led medicines information services describe hospital-based or regional models rather than statewide services, reported enquiry volumes are of a similar order of magnitude, particularly for specialist services managing high-risk or complex medication queries (Hands et al., 2002; McEntee et al., 2010; Rutter & Rutter, 2019). Direct comparison is limited by differences in service scope, catchment, and referral pathways. Uptake of PDAS is likely influenced by multiple factors, including those related to service visibility and awareness, highlighting opportunities for targeted outreach and education to support broader utilisation.

Enquiries most frequently concerned antipsychotics and antidepressants, with over half classified as moderate-to-high complexity. These reflect the breadth of clinical uncertainty faced by practitioners managing psychotropic medicines, and highlight PDAS’s role in supporting evidence-based, decision-making within a diverse mental health care landscape. Qualitative insights complemented these data by illustrating how PDAS extends beyond an informational function to fulfil a relational, collaborative, and confidence-building role in clinical practice.

These findings align with evidence demonstrating that pharmacist-led medicines information services improve decision quality, promote evidence-based practice, and enhance patient safety (Hands et al., 2002; McEntee et al., 2010; Bramley et al., 2019). In mental health, where prescribing decisions frequently involve uncertainty, off-label use, and risk–benefit trade-offs, PDAS represents a specialised extension of this model. (Prior, 2019; Campbell et al., 2021). Rutter and Rutter’s review (2019) reported that medicines information services play a key supportive role by helping clinicians validate decisions, manage risk, and maintain clarity in complex or high-stakes clinical situations. Participants described PDAS as a “second opinion,” indicating that the service provides not only specialist clinical information but also professional reassurance in situations where practitioners may feel isolated or unsupported. Importantly, the availability of PDAS to consumers and carers further extends its utility beyond traditional clinician-facing MI services, offering accessible, individualised guidance in circumstances where specialist input may otherwise be limited. Similar relational benefits were observed in a study of hospital pharmacy Medicines Helplines, where patients reported reductions in treatment uncertainty, improved understanding, and avoidance of potential harm following pharmacist advice (Bramley et al., 2019).

Quantitative findings demonstrate that PDAS responds to both lower-complexity psychotropic questions and more nuanced enquiries requiring specialist interpretation. While psychiatrists and pharmacists were frequent users, a substantial proportion of enquiries originated from other health professionals and from consumers and carers. Consumer enquiries frequently related to antipsychotic medicines, suggesting a particular need for support around treatments associated with serious adverse effects or severe mental illness. This breadth of users highlights PDAS’s role in supporting safe psychotropic decision-making across multiple care settings, including those where access to senior or specialist expertise may be constrained. The types of enquiries dealt with by the service ranged from administration and dosage, choice or selection of therapy and indications or contraindications, as well as information on drug–drug interactions and adverse effects. This is consistent with some of the findings reported in a study by Richardson et al. (2014), who similarly identified medication selection, dosing, drug interactions and adverse effect management as common drug-related problems addressed by pharmacists in inpatient mental health settings. Likewise, Ewan and Greene (2000) reported that antipsychotics were among the most frequently discussed medicines in a community-based pharmacist-run medication advice service, consistent with the proportion of antipsychotic-related enquiries observed in PDAS. However, unlike these earlier evaluations, which were largely confined to single-site inpatient or community settings, PDAS captured engagement across multiple sectors, including primary care, secondary care, and direct consumer and carer enquiries; highlighting its broader statewide consultative reach.

An important distinction highlighted by this evaluation is the interpretive nature of medicines information work in practice. As reflected in the UK Medicines Information (UKMi) guidance on ranking enquiries, complexity is independent of the time required to complete an enquiry. Experienced medicines information practitioners may reclassify apparently straightforward questions as more complex Level 2 or 3 enquiries once the full clinical context and implications are explored. Consistent with this, lower-complexity enquiries were significantly more likely to be completed within 30 minutes, whereas higher-complexity enquiries were more frequently associated with longer completion times (Table 3). The qualitative findings in this study further illustrate how seemingly simple questions concealed more nuanced clinical considerations. Through structured questioning, clinical judgement, and critical appraisal of the evidence, PDAS pharmacists frequently identified additional risks, contraindications, or treatment nuances requiring deeper evaluation. This interpretive function highlights the value of specialist medicines information services in supporting safe psychotropic prescribing beyond what can be achieved through static information resources alone.

International evidence demonstrates that medicines information needs range from routine, fact-based queries to highly complex, context-dependent clinical problems. Campbell et al. (2021) described this spectrum in general practice as comprising a ‘common core’ related to dosing, interactions and side effects and a ‘perplexing periphery’ characterised by multimorbidity, uncertainty and therapeutic ambiguity. Similar patterns have been reported in UK and European studies, where enquiries to medicines information and helpline services range from straightforward verification questions to complex scenarios requiring interpretation, and nuanced clinical judgement (McEntee et al., 2010; Rutter & Rutter, 2019; Hands et al., 2002; Bramley et al., 2019). The nature of enquiries to PDAS reflects this spectrum. Specific queries relating to dosage, substitutions, administration and adverse effects were common, but equally frequent were more complex issues such as co-occurring substance misuse, drug interactions, and drug effects in populations with specific co-morbidities or presenting complications, including pregnancy. Such questions are not easily addressed without specialist advice and consultation. Like the New Zealand general practice cohort, PDAS users faced barriers including limited time, information overload, and difficulty appraising conflicting sources. The breadth and depth of enquiries therefore reinforce the need for a specialist service capable of navigating this complexity, particularly in mental health.

In Victoria, Australia, statewide reform of the publicly funded mental health system is being driven by the Royal Commission into Victoria’s Mental Health System, which sets the policy direction for integrated, community-based service delivery across local, area-based, and specialist services (Royal Commission into Victoria’s Mental Health System, 2021*)*. The findings of this study indicate that PDAS aligns strongly with this reform agenda by operating as a statewide specialist service that provides accessible psychotropic medicines expertise across community and specialist mental health settings, consistent with the Royal Commission’s emphasis on integration, accessibility, and contemporary models of care.

The nature of enquiries received by PDAS also provides insight into emerging psychotropic practice challenges, including areas of persistent uncertainty, safety concerns, and unmet informational needs. This pattern reflects the increasing complexity of mental health care, and the difficulty clinicians face in applying psychotropic evidence in real-world settings. By capturing these trends, PDAS contributes not only to individual clinical decision-making but also to a broader understanding of system-level needs, with implications for guideline development, education priorities, and service planning. While these findings offer valuable insights, several methodological considerations warrant attention. As reported in previous studies, it is important that services that offer advice on psychotropic medications are run by pharmacists with psychiatric experience. In their study, Ewan and Greene (2000) reported that community pharmacists found it difficult at times to answer psychotropic related enquiries and needed to refer such patient enquiries to specialist psychiatric pharmacists who have expertise in the area.

Advances in digital decision support tools, including the increasing use of artificial intelligence (AI), are likely to influence how medicines information is accessed and utilised within healthcare systems. While AI-enabled tools may assist with retrieval of standardised information and support lower-complexity queries, findings from this evaluation suggest that specialist clinical medicines information services continue to play a critical role. A substantial proportion of enquiries, including those classified as lower complexity, still required interpretation within a mental health context, application of clinical judgement, and consideration of nuanced patient-specific factors. Furthermore, many enquiries involved uncertainty, distress, or system navigation that cannot be readily addressed by automated tools alone. Rather than replacing specialist services, emerging digital and/or AI based tools may complement clinical medicines information services such as PDAS by supporting information retrieval, allowing specialist clinicians to focus on interpretation, supported decision-making, and patient-centred care.

## Conclusion

This evaluation affirms that PDAS contributes meaningfully to safe, informed, and person-centred psychotropic prescribing and medication management. The four key themes identified—enhancing confidence in decision-making, improving access to specialist advice, supporting collaborative clinical reasoning, and delivering credible evidence-based guidance—illustrate the multifaceted value of the service. PDAS operates not only as a trusted source of psychopharmacological expertise but also as an enabling mechanism that supports reflective practice, interdisciplinary learning, and shared decision-making with consumers and carers.

Future research could examine the longitudinal clinical, safety, and economic impacts of PDAS, including its influence on treatment optimisation, adverse event prevention, and consumer outcomes. Strengthening digital integration—such as embedding PDAS guidance within statewide prescribing or clinical decision-support tools—may further enhance accessibility and consistency. Ongoing engagement with clinicians, consumers, and carers will be essential to ensure the service remains responsive to evolving needs and continues to foster strong, trust-based relationships with its user base. As demand for the service grows, strategic workforce expansion and deeper cross-sector partnerships may position PDAS as a central pillar of statewide psychotropic stewardship and quality use of medicines. Finally, improving the visibility and promotion of PDAS at a system level will be essential to ensuring clinicians, consumers, and carers are aware service’s availability, scope, and role; enabling broader uptake and sustained impact.

### Limitations

This evaluation has several strengths, including its mixed-methods design, the integration of quantitative service-use data with rich qualitative insights, and the inclusion of diverse stakeholder perspectives spanning psychiatrists, pharmacists, general practitioners, consumers, and carers. The incorporation of consumer and carer voices is a particular strength, offering insight into how specialised psychotropic medicines information services are experienced by those directly impacted. However, several limitations should be acknowledged. The qualitative sample size was modest and may not capture the full diversity of PDAS users, raising the possibility of response bias, particularly as participation was voluntary and may have attracted individuals with more positive or engaged experiences. Quantitative findings were also limited by reliance on data from a single jurisdiction (Victoria) which may constrain generalisability to settings with different service structures or referral pathways. In addition, without longitudinal follow-up or objective outcome measures, findings primarily reflect service utilisation and perceived impact rather than effectiveness. Finally, as with all retrospective service evaluations, findings may have been influenced by variations in documentation quality or how enquiries were recorded. These limitations should be considered when interpreting the results and highlight opportunities for future research, including larger multi-site evaluations and longitudinal assessments of clinical impact.

## Data Availability

The interview data from this study cannot be shared openly to protect study participant privacy.
